# Microbiota from alginate oligosaccharide-dosed mice successfully mitigated small intestinal mucositis

**DOI:** 10.1186/s40168-020-00886-x

**Published:** 2020-07-25

**Authors:** Pengfei Zhang, Jing Liu, Bohui Xiong, Cong Zhang, Beining Kang, Yishan Gao, Zengkuan Li, Wei Ge, Shunfeng Cheng, Yanan Hao, Wei Shen, Shuai Yu, Liang Chen, Xiangfang Tang, Yong Zhao, Hongfu Zhang

**Affiliations:** 1 State Key Laboratory of Animal Nutrition, Institute of Animal Sciences, Chinese Academy of Agricultural Sciences, Beijing, 100193 People’s Republic of China; 2 College of Life Sciences, Qingdao Agricultural University, Qingdao, 266109 People’s Republic of China; 3 University Research Core, Qingdao Agricultural University, Qingdao, 266109 People’s Republic of China; 4 College of Animal Sciences and Technology, Qingdao Agricultural University, Qingdao, 266109 People’s Republic of China; 5 Center for Reproductive Medicine, Urology Department, Peking University Shenzhen Hospital, Shenzhen, 518036 People’s Republic of China

**Keywords:** Fecal microbiota transplantation, Alginate oligosaccharides, Mucositis, Busulfan, Rescue, Metabolome, Correlation

## Abstract

**Background:**

The increasing incidence of cancer and intestinal mucositis induced by chemotherapeutics are causing worldwide concern. Many approaches such as fecal microbiota transplantation (FMT) have been used to minimize mucositis. However, it is still unknown whether FMT from a donor with beneficial gut microbiota results in more effective intestinal function in the recipient. Recently, we found that alginate oligosaccharides (AOS) benefit murine gut microbiota through increasing “beneficial” microbes to rescue busulfan induced mucositis.

**Results:**

In the current investigation, FMT from AOS-dosed mice improved small intestine function over FMT from control mice through the recovery of gene expression and an increase in the levels of cell junction proteins. FMT from AOS-dosed mice showed superior benefits over FMT from control mice on recipient gut microbiotas through an increase in “beneficial” microbes such as *Leuconostocaceae* and recovery in blood metabolome. Furthermore, the correlation of gut microbiota and blood metabolites suggested that the “beneficial” microbe *Lactobacillales* helped with the recovery of blood metabolites, while the “harmful” microbe *Mycoplasmatales* did not.

**Conclusion:**

The data confirm our hypothesis that FMT from a donor with superior microbes leads to a more profound recovery of small intestinal function. We propose that gut microbiota from naturally produced AOS-treated donor may be used to prevent small intestinal mucositis induced by chemotherapeutics or other factors in recipients.

Video Abstract

## Introduction

The annual incidence of cancer is continuing to increase and cause worldwide concern and frustration [1–4]. Furthermore, intestinal mucositis is an adverse effect of chemotherapy with anticancer drugs such as busulfan, 5-fluorouracil [5–7], or FOLFOX (5-fluorouracil, leucovorin, and oxaliplatin) [8]. It is reported that villi length is reduced and crypt cell homeostasis and intestinal tight junctions are impaired in intestinal mucositis [2, 4]. Since the gastrointestinal (GI) tract plays such vital roles—protection of the body from pathogenic microbes, nutrient digestion/absorption, mucus, and hormone secretion—mucositis can cause clinical morbidity and mortality [5–7, 9–11]. Even though lots of studies have addressed many methods such as the application of prebiotics, probiotics, selenium, volatile oils, and others [1, 2, 12, 13] to minimize chemotherapy-induced gut mucositis, there has been little progress [14, 15]. Alginate oligosaccharides (AOS) are natural products derived from the degradation of alginate that have attractive pharmaceutical properties [16–18]. They have been found to be anti-inflammatory [17], anti-apoptotic [19], anti-proliferation [20], and to have antioxidant [16, 19, 21] and even anti-cancer activities [22]. Very recently, we showed that AOS rescues busulfan disrupted murine small intestinal cell endoplasmic reticulum and mitochondria [23]. Furthermore, AOS improves busulfan disturbed intestinal cell membranes through the enhancement of cell junctions, recovery of small intestinal functions (as shown by the single-cell RNA-seq analysis), and improvement of transcriptional factors which may contribute to the gene expression [23]. Furthermore, AOS improves the blood metabolome to support the recovery of small intestine function [23]. Subsequently, we also found that AOS may benefit gut microbiota through an increase in “beneficial” gut microbes and a decrease in “harmful” gut microbes (unpublished data). In addition, Chang et al. recently found that fecal microbiota transplantation (FMT) prevents FOLFOX-induced intestinal mucositis [8].

Gut microbiota is reported to influence many aspects of our health because it provides nutrients and vitamins, fights against pathogens, maintains homeostasis of the epithelial mucosa, and supports the body’s immune system [24]. On the other hand, microbiota dysbiosis has been shown to lead to various diseases [25, 26] such as diabetes, hypertension, IBD (inflammatory bowel disease), obesity, polycystic ovary syndrome (PCOS) [27], and disruption of spermatogenesis [28]. FMT is the last chance treatment for *Clostridium difficile* infections [25, 26], and it also has been applied in many disease models and clinical trials with a very high cure rate and few adverse effects [29, 30]. FMT has been revealed to effectively manipulate gut microbiota and to ameliorate chemotherapy-induced mucositis [8, 31], to relieve mouse Parkinson’s disease [32], to treat food allergies [33], and to increase healthspan and lifespan [34]. Regular FMT involves the transplantation of fecal material from a healthy donor into a diseased recipient [8]. However, we found that AOS also rescues busulfan-impaired gut microbiota in the small intestine [23]. We hypothesize that FMT from AOS-dosed mice might alleviate small intestine disruption following recipient chemotherapy to a greater extent than FMT from control animals. Indeed, we found that gut microbiota from AOS-dosed mice was superior in improving gut microbiota, the blood metabolome, and the small intestine function.

## Results

### AOS benefited gut microbiota

**Fig. 1:**
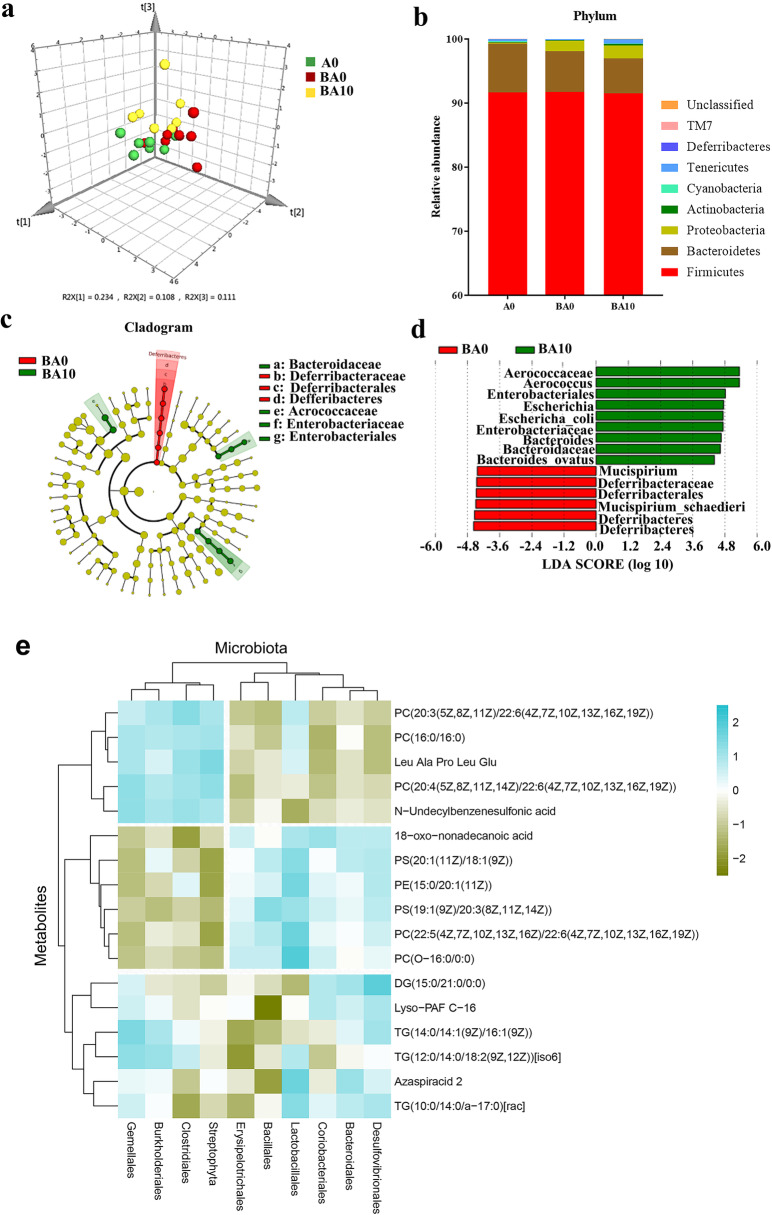
Small intestinal microbiota changes and correlation of changed intestinal microbiota after a 2-week AOS dosing. **a** The PLS-DA of the microflora in different treatments. **b** Differences of bacterial abundance at the phylum level. **c** Cladogram. **d** LDA distribution. Linear discriminate analysis effect size (LEfSe) was performed to determine the difference in abundance; the threshold of LDA score was 4.0 (*n* = 15 samples/group). **e** Correlation of intestinal microbiota and blood metabolites. (*n* = 10 samples/group)

In a recent report, we revealed that AOS mitigated small intestine cell membranes damage especially cell junctions and microvilli by the anticancer drug busulfan; simultaneously, AOS supported the blood metabolome to assist small intestinal recovery [23]. This is because gut microbiota can metabolize nutrients and also regulate intestinal metabolites to affect the blood metabolome [35, 36]. Therefore, in the current investigation, we explored changes in gut microbiota after 2 weeks of busulfan and/or AOS treatment [there were three treatment groups: (1) A0: vehicle control (dosed with ddH_2_O); (2) BA0 [injected with 40 mg/kg body weight (BW) busulfan once then dosed with ddH_2_O]; (3) BA10 (injected with 40 mg/kg BW busulfan once then dosed with 10 mg/kg BW AOS in ddH_2_O for 2 weeks continually). The differences in bacterial composition between the three treatments were clearly separated by weighted principal coordinates analysis (PCoA; Fig. 1a; Supplementary Table 1; Supplementary Fig. 1). At the genus level, the proportion of *Lactobacillus* was increased by BA10 compared with A0 or BA0 (Supplementary Table 1), even though at the phylum level, there was little difference in the proportion of the bacteria (Fig. 1b). Then, the linear discriminant analysis effect size (LEFSe) was used to further evaluate the difference in bacterial content between these treatments (Fig. 1c, d). *Bacteroidaceae* was enriched in BA10, but not in other groups, which indicated that AOS benefited the gut microbiota by increasing “beneficial” gut microbiota (Fig. 1c, d). Even though the harmful gut microbiota *Escherichia* and *E. coli* were also enriched in BA10, this may be due to busulfan effect and the relative significance was low. Another of our studies [37] shows that AOS increases the “beneficial” gut bacteria such as *Bacteroidales* and *Lactobacillaceae* while it decreases “harmful” bacteria *Desulfovibrionaceae* after a 5-week AOS dosing period (in the current study, the treatment time was 2 weeks). The data from these two studies were in agreement, which indicates that AOS benefits gut microbiota.

At the same time, AOS benefits blood metabolites [23]; therefore, the crosstalk between blood metabolites and gut microbes was determined by Spearman’s correlation coefficient, which reflects the correlation of blood metabolites and gut microbiota [38]. Here, there was a good correlation between blood metabolome and gut microbiota (Fig. 1e). The microbes were divided into two big clusters (left and right) and the metabolites were separated into three big clusters (top, middle, and bottom). The first cluster of microbes (left) and the first cluster of metabolites (top) were positively correlated together, while the second cluster of microbes (right) was positively correlated with the second cluster of metabolites (middle). *Lactobacillales* was positively correlated with most of the metabolites (Fig. 1e). The data suggested that the blood metabolome and gut microbiota interacted together, which further indicated that AOS treatment benefited gut microbiota to improve the small intestine function.

### A10-FMT/A100-FMT improved small intestine function more profoundly than Con-FMT

**Fig. 2:**
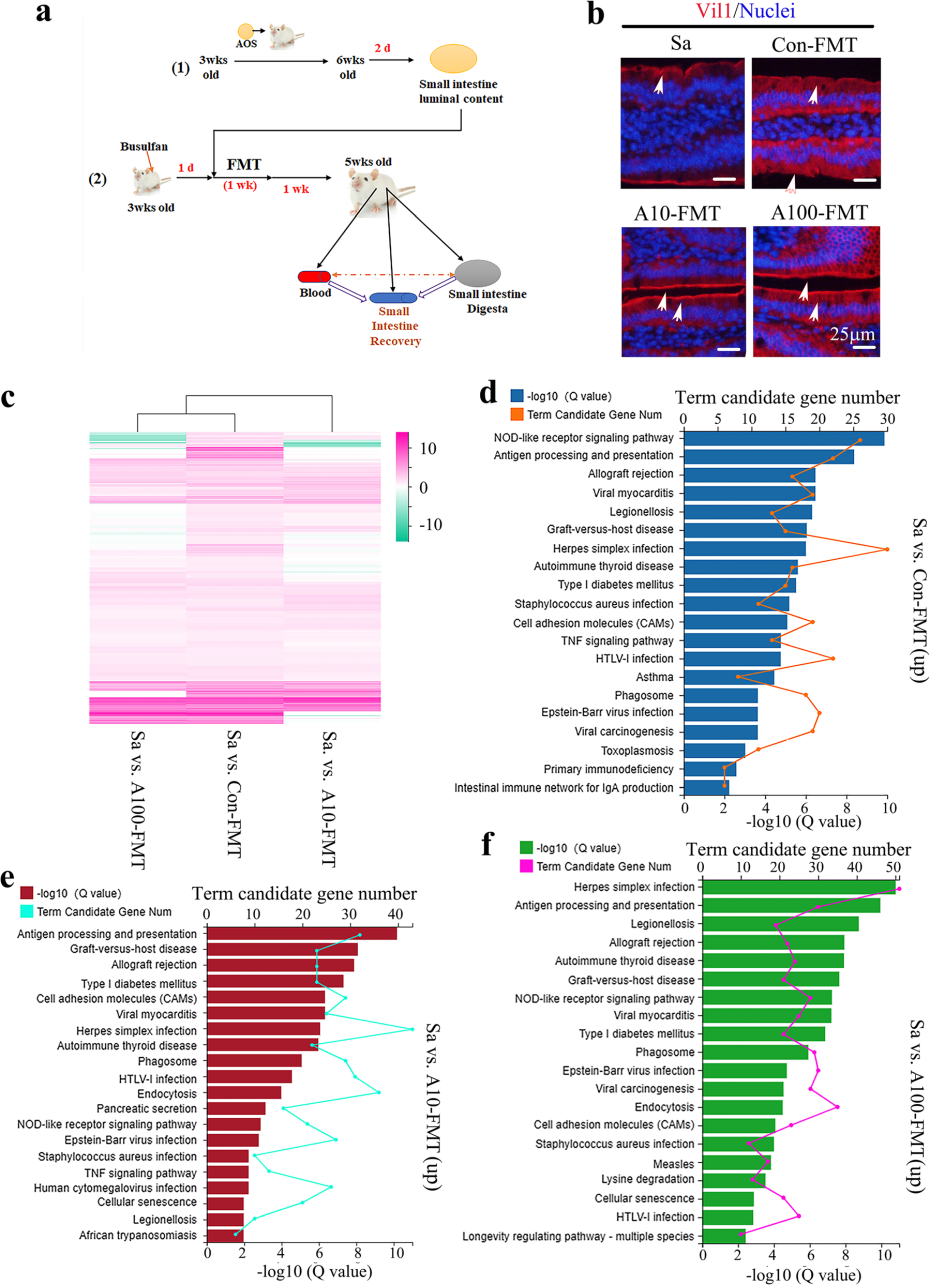
RNA-seq data for mouse small intestine samples. **a** Study design for the FMT experiment. Firstly, 3-week-old ICR male mice were treated with ddH_2_O as the vehicle control or AOS [10 or 100 mg/kg body weight (BW)] for 3 weeks. Then the mice were maintained in regular condition for another 2 days (with dosing). Then the mice were humanely euthanized to collect small intestine luminal contents (gut microbiota). The luminal contents from each group were pooled and homogenized, diluted 1:1 in 20% sterile glycerol (saline), and frozen. Before inoculation, small intestinal content samples were diluted in sterile saline to a working concentration of 0.05 g/ml and filtered through a 70-μm cell strainer (FMT). Secondly, 3-week-old ICR male mice were injected a single dose of busulfan [40 mg/kg body weight (BW)] [23]. The following day, the mice were dosed with saline as the control or FMT via oral gavage (0.1 ml/mouse/day). Recipient mice received oral FMT inoculations once daily for 1 week. The mice were then regularly maintained for another week (5 weeks of age) and then humanely euthanized to collect samples for different analyses. **b** Immunofluorescence staining of Vil1 for small intestine samples. **c** Heatmap summary of the differentially expressed genes in the three comparisons: Sa vs. Con-FMT; Sa vs. A10-FMT; Sa vs. A100-FMT. The scale bar shows the gene expression in each group. The clusters show the groups of genes in a similar gene family. **d** KEGG enrichment of up-regulated genes in Sa vs. Con-FMT. **e** KEGG enrichment of up-regulated genes in Sa vs. A10-FMT. **f** KEGG enrichment of up-regulated genes in Sa vs. A100-FMT

Since AOS benefited gut microbiota to improve the busulfan disrupted small intestine, we set out to explore the beneficial improvement of small intestinal functions by FMT from AOS 10 mg/kg, 100 mg/kg, and vehicle control-dosed mice. As shown in Fig. 2a, gut microbiota (intestinal luminal content) [39] was collected from AOS (10 mg/kg, or 100 mg/kg) or ddH_2_O (vehicle control)-dosed mice, respectively. The gut microbiota was diluted in saline for FMT. There were four treatment groups (see “Methods” section). We found that the busulfan “Sa” group (mice treated with busulfan and saline) impaired the small intestine by reducing the density of microvilli compared with the control group (mice treated with saline only; Supplementary Fig. 2a) and that Con-FMT (mice treated with busulfan and FMT from the vehicle control group) had some effect on small intestinal improvement (Fig. 2b). Meanwhile, A10-FMT (mice treated with busulfan and FMT from the AOS 10 mg/kg group) and A100-FMT (mice treated with busulfan and FMT from the AOS 100 mg/kg group) dramatically recovered the small intestine by increasing the protein levels of Vil1 (Fig. 2b). The results matched the data from our earlier study, showing that AOS and A10-FMT/A100-FMT assisted in the recovery of the small intestine by increasing Vil1 protein levels [23].

To explore the underlying mechanisms of the A10-FMT/A100-FMT improvement of small intestines, gene expression profiles for mouse small intestine were quantified by RNA-seq analysis. The gene expression profiles were changed significantly by Con-FMT, A10-FMT, and A100-FMT. In total, 166 genes were decreased while 308 genes were increased by Con-FMT compared with Sa (Sa vs. Con-FMT); 179 genes were reduced while 540 genes were elevated by A10-FMT compared with Sa (Sa vs. A10-FMT); 267 genes were diminished while 572 genes were increased by A100-FMT compared with Sa (Sa vs. A100-FMT; Fig. 2c and Supplementary Fig. 2b–h). The functions of the altered genes were determined by Kyoto Encyclopedia of Genes and Genomes (KEGG) pathway analysis. The most commonly enriched pathways in these three comparisons (Sa vs. Con-FMT; Sa vs. A10-FMT; Sa vs. A100-FMT) were NOD-like receptor signaling pathway, antigen processing and presentation, and cell adhesion molecules (CAMs). The pathways “NOD-like receptor signaling pathway” and “antigen processing and presentation” are related to immune functions, which suggested that FMT may benefit immune function in murine intestines (Fig. 2d–f). The pathway “cell adhesion molecules” was more significantly enriched in A10-FMT than Con-FMT, this indicated that cell junction improvement was more profound in A10-FMT, which is reflected by the following results (Fig. 2d–f). There were a few pathways enriched in A10-FMT/A100-FMT but not in Con-FMT such as endocytosis which indicated that A10-FMT/A100-FMT more beneficially improved small intestine function (Fig. 2d–f). On the other hand, the decreased genes in these three comparisons were enriched into functional pathways dissimilar to those in the increased genes, and the significance of the enrichment was lower (Supplementary Fig.2i-k).

**Fig. 3:**
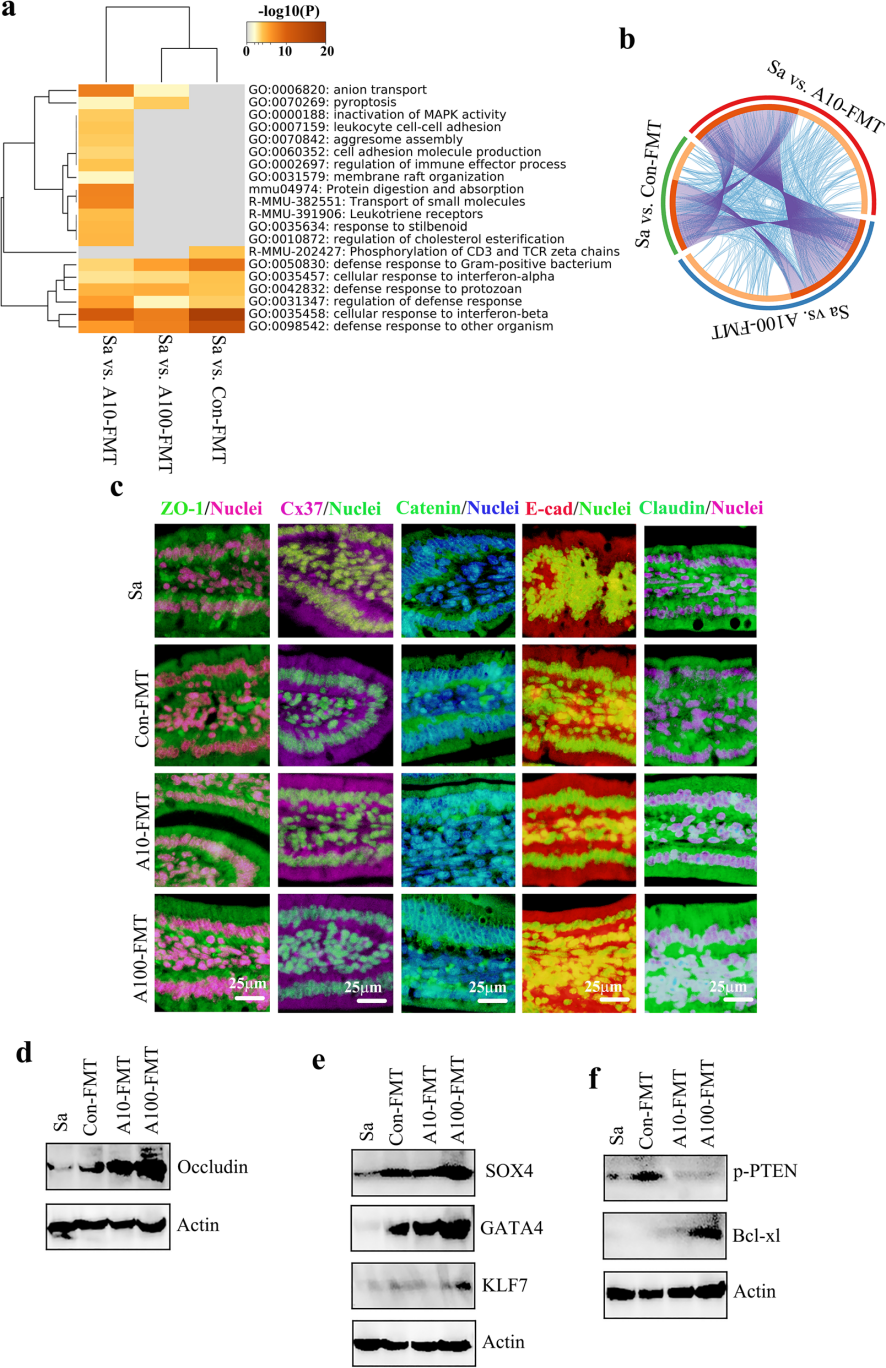
Multiple enrichment analysis and cell junction protein levels. **a** Multiple enrichment analysis for the increased genes in Sa vs. Con-FMT, Sa vs. A10-FMT, and Sa vs. A100-FMT using the online tool in Metascape. **b** Circos plots showing an interaction between these comparisons. The shared marker genes are linked by purple lines, and similar terms are linked by blue lines. **c** Immunofluorescence staining (IHF) for some of the cell junction molecules in murine small intestines. **d** Western blotting analysis of cell junction protein occludin in small intestine samples. **e** Western blotting analysis of transcriptional factors in small intestine samples. **f**, Western blotting analysis of p-PTEN and Bcl-xl in small intestine samples

Furthermore, the increased genes from the three comparisons (Sa vs. Con-FMT, Sa vs. A10-FMT, A100-FMT) were determined by multiple enrichment analysis (Metascape: http://metascape.org/gp/index.html#/ main/step1). Results showed that the common functional groups were related to cell defense (Fig. 3a, b). The most specific functional groups were enriched in Sa vs. A10-FMT, including cell adhesion molecules, digestion, and absorption which reflected the recovery of the small intestine (Fig. 3a). The results suggested that the small intestine was improved by A10-FMT more profoundly, and as we reported recently, AOS 10 mg/kg was the best concentration for this improvement [23].

Since “CAM” was enriched in the RNA-seq data, the important cell adhesion molecules (cell junction proteins) were determined in the small intestine samples. Protein levels of important cell junction molecules ZO-1, Cx37, Catenin, E-cad (E-cadherin), and claudin were lowest in the Sa group, higher in Con-FMT, and highest in A10-FMT and A100-FMT (Fig. 3c), which suggested that A10-FMT and A100-FMT had a more profound effect on small intestine improvement. The data were confirmed by Western blotting analysis for occludin (Fig. 3d). In our earlier study, we found that AOS improved transcriptional factors in the small intestine. In the current investigation, the protein levels of transcriptional factors SOX4, GATA4, and KLF7 were lowest in Sa, while highest in A100-FMT (Fig. 3e). At the same time, the level of apoptosis was higher in Sa and Con-FMT while lower in A10-FMT and A100-FMT, as indicated by the protein levels of p-PTEN and Bcl-xl (Fig. 3f). All the data in this section strongly suggested that A10-FMT and A100-FMT were more beneficial to the busulfan-impaired small intestine than Con-FMT, and the data matched well with our earlier study [23].

### A10-FMT/A100-FMT benefited gut microbiota more profoundly than Con-FMT

**Fig. 4:**
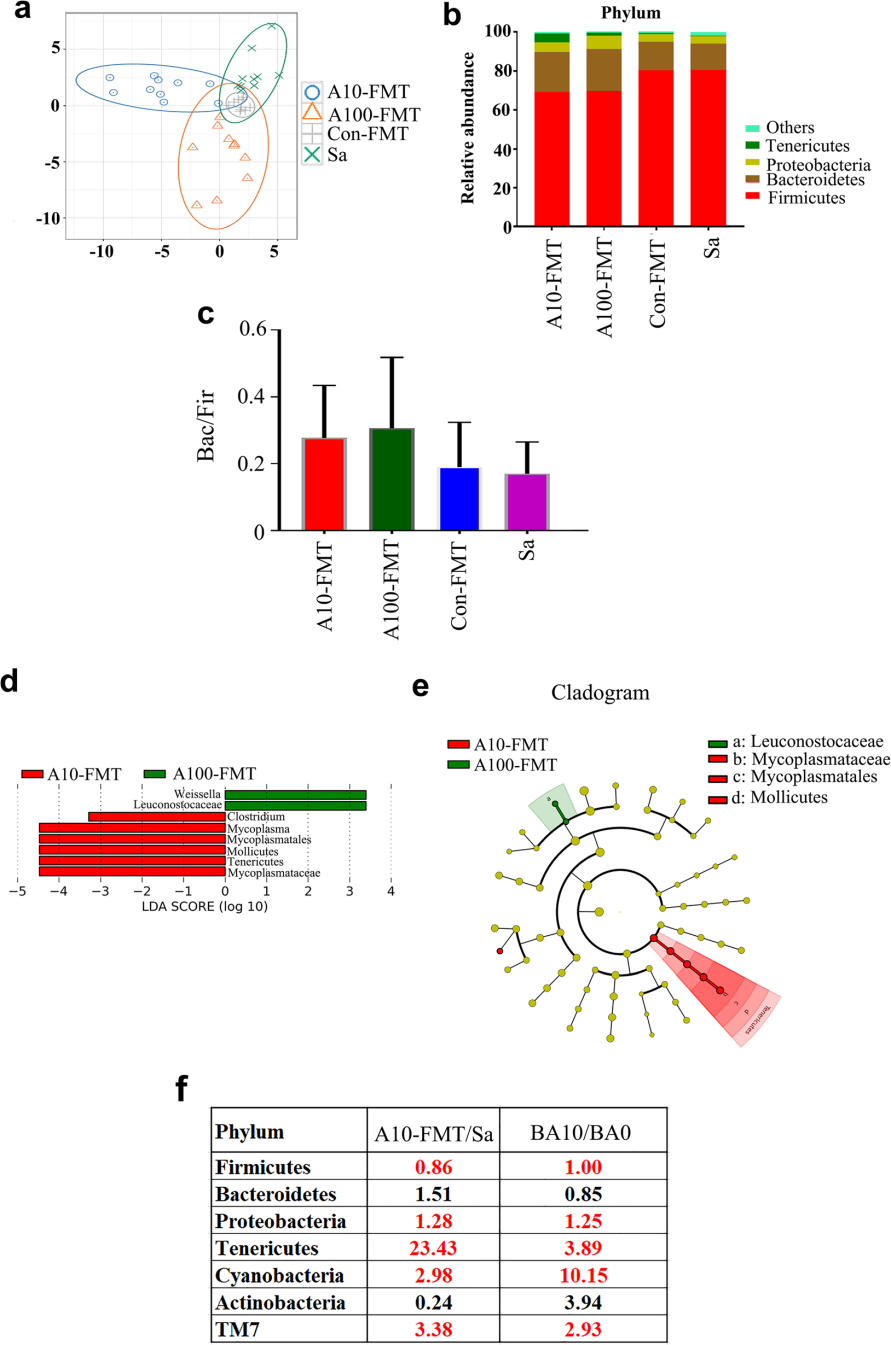
Changes in small intestinal microbiota after FMT treatment. **a** The PLS-DA of the microflora in different treatments. **b** Differences of bacterial abundance at the phylum level. **c** The ratio of *Bacteriodetes* to *Firmicutes*. **d** LDA distribution. **e**, Cladogram. Linear discriminate analysis effect size (LEfSe) was performed to determine the difference in abundance; the threshold of LDA score was 4.0 (*n* = 15 samples/group). **f** Correlation of intestinal microbiota after FMT treatment and gut microbiota after 2-weeks of AOS treatment

To explore small intestine improvement by FMT through the gut microbiota, gut microbial proportions were determined by 16S-rDNA sequencing analysis (Supplementary Fig. 3; Supplementary Table 2). The differences in bacterial composition between the four treatment groups were separated by weighted PCoA; however, group Sa and Con-FMT were not well separated (Fig. 4a). Levels of the “beneficial” bacteria *Bacteroidetes* were higher in the A10-FMT and A100-FMT groups, while the “harmful” bacteria *Firmicutes* was elevated in the Sa and Con-FMT groups (Fig. 4b). Moreover, the “harmful” bacteria *Akkermancia* was present just in Sa group. Furthermore, the ratio of *Bacteroidetes*/*Firmicutes* was higher in A10-FMT and A100-FMT than that in Sa or Con-FMT even though the difference was not significant (Fig. 4c), which indicated that A10-FMT and A100-FMT benefited gut microbiota more profoundly than Con-FMT. LEFSe was performed to further explore the difference in bacterial content between the four groups (Fig. 4d, e). *Leuconostocaceae* were enriched in A100-FMT while not in other groups, which suggested that A100-FMT increased the beneficial bacteria as we found in our earlier study [23].

The gut microbiotas in the FMT study and the AOS direct treatment study were determined to search for correlations between these two studies. The ratios of A10-FMT/Sa in the FMT study and BA10/BA0 in the AOS direct treatment study showed similar trends for most microbiotas at the “phylum” level (Fig. 4f), which suggested the microbes in these two studies were well correlated.

### A10-FMT/A100-FMT recovered the blood metabolome more profoundly than Con-FMT

**Fig. 5:**
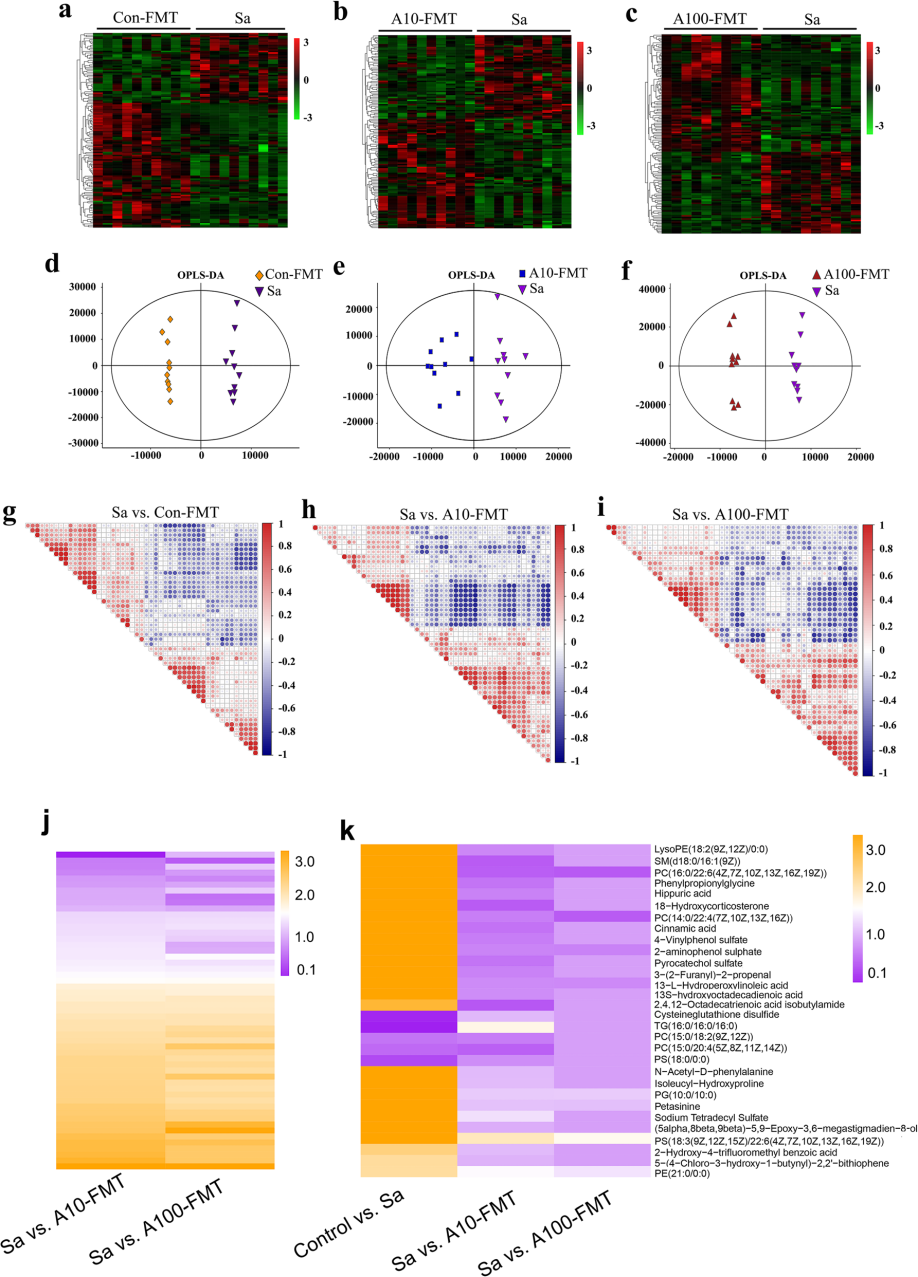
Blood metabolome changes. **a** Heatmap of changed blood metabolites in Sa and Con-FMT. **b** Heatmap of changed blood metabolites in Sa and A10-FMT. **c** Heatmap of changed blood metabolites in Sa and A100-FMT. **d** PLS-DA of murine blood metabolites in the Sa and Con-FMT groups. **e** PLS-DA of murine blood metabolites in the Sa and A10-FMT groups. **f** PLS-DA of murine blood metabolites in the Sa and A100-FMT groups. **g** Correlation of the metabolites in Sa vs. Con-FMT. **h** Correlation of the metabolites in Sa vs. A10-FMT. **i** Correlation of the metabolites in Sa vs. A100-FMT. **j** Heatmap of commonly changed blood metabolites in Sa vs. A10-FMT and Sa vs. A100-FMT. **k** Heatmap of commonly changed blood metabolites in control vs. Sa, Sa vs. A10-FMT, and Sa vs. A100-FMT

Gut microbiota plays vital roles in nutrient digestion and absorption to influence blood metabolism [38]. Next, we set out to explore the effects of FMT on the blood metabolome using ultra-performance liquid chromatography-coupled time-of-flight mass spectrometry (UPLC-ESI-QTOFMS) and examine the correlation between blood metabolism and the gut microbiota. Con-FMT, A10-FMT, and A100-FMT significantly changed blood metabolites (Fig. 5a–c). The plot from the partial least squares discriminant analysis (PLS-DA) clearly presents the differences in metabolite composition between Sa and Con-FMT, Sa and A10-FMT, and Sa and A100-FMT (Fig. 5d–f; Supplementary Fig. 4a). There were 131, 132, and 129 significantly changed metabolites (positive and negative modes) for the following comparisons: Sa vs. Con-FMT, Sa vs. A10-FMT, and Sa vs. A100-FMT, respectively (Data file 1). The metabolites in each comparison were correlated with each other (Fig. 5 g–i; Supplementary Fig. 4). It was interesting to notice that the 53 metabolites common for Sa vs. A10-FMT and Sa vs. A100-FMT had similar trends (Fig. 5j; Supplementary Table 3), which suggested that A10-FMT and A100-FMT influenced blood metabolites in the same manner. Thirty metabolites were common for the comparisons control vs. Sa, Sa vs. A10-FMT, and Sa vs. A100-FMT, while no metabolites from Sa vs. Con-FMT were common with the latter 30 metabolites. Moreover, 15 out of the 30 metabolites were increased by busulfan (in control vs. Sa) while they were decreased by A10-FMT and A100-FMT (in Sa vs. A10-FMT and Sa vs. A100-FMT; Fig. 5 k). In addition, two out of the 30 metabolites were decreased by busulfan (in control vs. Sa) while they were increased by A10-FMT and A100-FMT (in Sa vs. A10-FMT, Sa vs. A100-FMT; Fig. 5k), which suggested that A10-FMT and A100-FMT produced a superior improvement in the blood metabolome. Most of these 30 metabolites were lipid-like molecules that play very important roles in small intestine function, which indicated that A10-FMT/A100-FMT recovered those metabolites that were upset by busulfan in the blood.

**Fig. 6:**
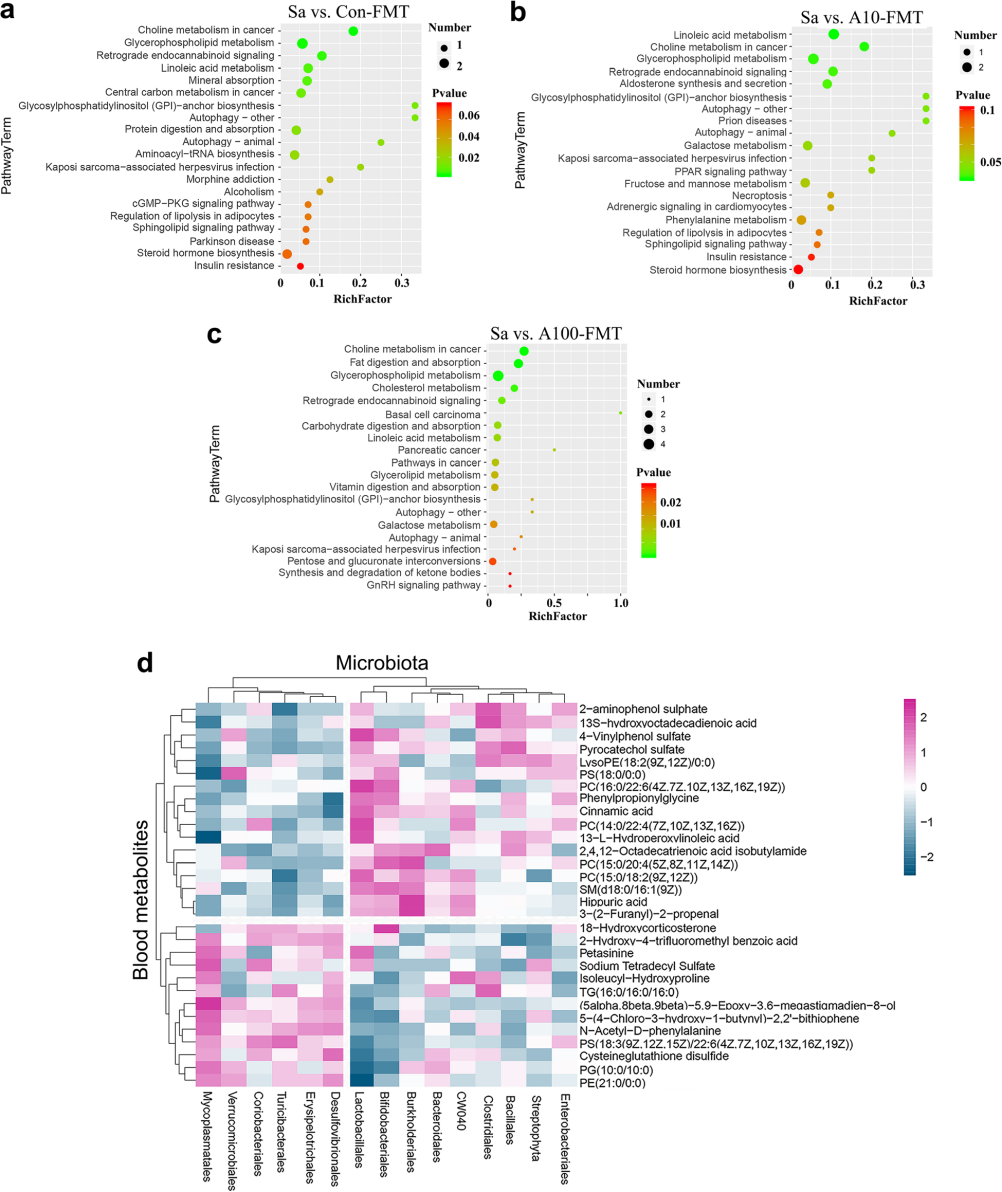
Correlations of blood metabolome and gut microbiota. **a** KEGG enriched pathways of changed blood metabolites in Sa vs. Con-FMT. **b** KEGG enriched pathways of changed blood metabolites in Sa vs. A10-FMT. **c** KEGG enriched pathways of changed blood metabolites in Sa vs. A100-FMT. **d** Correlation of blood metabolites and gut microbiota

The functions of these changed metabolites were determined by KEGG pathway analysis. The common pathways enriched in the three comparisons (Sa vs. Con-FMT; Sa vs. A10-FMT; Sa vs. A100-FMT) included choline metabolism in cancer, glycerophospholipid metabolism, retrograde endocannabinoid signaling, linoleic acid metabolism, and glycosylphosphatidylinositol (GPI) anchor biosynthesis (Fig. 6a–c). The specific pathways enriched in Sa vs. A10-FMT were aldosterone synthesis and secretion, PPAR signaling pathways, fructose and mannose metabolism, and galactose metabolism. The specific pathways enriched in A100-FMT included fat digestion and absorption, cholesterol metabolism, carbohydrate digestion and absorption, and vitamin digestion and absorption (Fig. 6a–c). Although there were some different pathways in the comparison of Sa vs. A10-FMT and Sa vs. A100-FMT, they had lots of pathways in common. And these pathways reflected the benefit advantages of A10-FMT and A100-FMT in the improvement of the small intestine function. The data herein suggested that A10-FMT and A100-FMT were superior to Con-FMT for improving blood metabolites.

The correlation of blood metabolites and gut microbiota was analyzed by Spearman’s correlation coefficient (Fig. 6d). The blood metabolites were divided into two clusters (up and down; Fig. 6d). Most of the metabolites in the up cluster were increased by Sa (control vs. Sa) while decreased by A10-FMT and A100-FMT. Most of the metabolites in the down cluster had the same trend in control vs. Sa, Sa vs. A10-FMT and Sa vs. A100-FMT. The microbiotas were also separated into two big clusters (left and right; Fig. 6d). The metabolites in the up cluster were positively correlated with the microbes in the right cluster, while the metabolites in the down cluster were positively correlated with the microbes in the left cluster (Fig. 6d). The most correlated microbes with blood metabolites were *Lactobacillales* and *Mycoplasmatales* (Fig. 6d). *Lactobacillales* was positively correlated with the up cluster metabolites (Fig. 6d) which suggested that the “beneficial microbe” *Lactobacillales* assisted in the recovery of blood metabolites because they reflect the normal small intestinal function [23]. Meanwhile, *Mycoplasmatales* was positively correlated with the down cluster metabolites (Fig. 6d), which suggested that the “harmful microbe” *Mycoplasmatales* was not able to assist in the recovery of blood metabolites [23]. The data suggested that A10-FMT and A100-FMT improved small intestine function and gut microbiota to recover the blood metabolome.

## Discussion

The intestine possesses the largest mucosal surface of the body; it produces mucins, hormones, and antimicrobial molecules to build a physical and chemical barrier to protect the body against pathogenic microbes; and it also digests and absorbs nutrients into the blood [9, 40]. Moreover, tight junction proteins (TJs) and the proteins related to cell renewal play important roles in maintaining the gut barrier [41]. Furthermore, gut microbiota plays a vital role in the regulation and function of the intestinal barrier [42], and disturbance of gut microbiota may lead to the disruption of the gut barrier to cause multiple diseases [43, 44]. Since FMT is successful in the treatment of *Clostridium difficile* infection (CDI) and inflammatory bowel disease (IBD), great interest has been bolstered to investigate its potential applications for other diseases [31, 32, 35]. Regular FMT involves transferring microbiota from healthy donors to recipients (patients) to modify their gut microbiota to cure diseases [31, 32, 35]. However, it is unknown whether the FMT from a donor effectively functions in a recipient. In our recent study, we found that a 5-week AOS dosing program benefited murine gut microbiota through an increase in “beneficial” microbes such as *Bacteroidales* and *Lactobacillaceae* and a decrease in “harmful” bacteria, such as *Desulfovibrionaceae* [37]. Moreover, in the current investigation, we found that a 2-week AOS dosing program also improved gut microbiota through an increase in “beneficial” microbes such as *Bacteroidales* and *Lactobacillaceae*. The gut microbiota and blood metabolites were also well correlated, which suggested that AOS benefited gut microbiota to heal the small intestine and then recover blood metabolites. Furthermore, FMT from AOS-dosed mice rescued the busulfan-disrupted small intestine, benefited gut microbiota, and recovered blood metabolites more profoundly than FMT from control mice (no AOS dosing). This is the first study to demonstrate that FMT from a donor with desirable microbiota produced superior results to FMT from a control donor in the improvement of small intestinal function.

FMT from AOS-dosed mice produced more profound improvements over FMT from control mice on small intestine function through the recovery of the expression of those genes involved in immune functions, cell junctions, and nutrient digestion and absorption. There was a small difference for A10-FMT and A100-FMT, although overall A10-FMT produced a more profound improvement than A100-FMT. Multiple functional enrichment analysis showed that those genes increased by A10-FMT were enriched into more functional pathways related to normal intestinal functions. At the same time, FMT from AOS-dosed mice were more effective than FMT from control mice in increasing the protein levels of cell junction proteins ZO-1, Cx37, catenin, E-cadherin, claudin, and occludin, which are important for maintaining the small intestine barrier and functions [8, 23, 31]. The data suggested that FMT from AOS-dosed mice rescued small intestine function in a similar manner as AOS did [23]. FMT from AOS-dosed mice showed a more profound benefit on gut microbiota than FMT from control mice, through an increase in the “beneficial” microbe *Leuconostocaceae*. This data matched data from AOS-dosed mice with an increase in “beneficial” microbes such as *Bacteroidales* and *Lactobacillaceae*. Moreover, the proportion of gut microbiota in A10-FMT/Sa was similar to that in BA10/BA0. Nutrient digestion and absorption take place in the small intestine [9, 40]; thus, the improved small intestine may reflect an improvement in the blood metabolome. Furthermore, FMT from AOS-dosed mice was more successful in recovering the blood metabolome than FMT from control mice. FMT from AOS-dosed mice also decreased the levels of busulfan-increased metabolites. On the other hand, FMT from AOS-dosed mice increased the levels of busulfan-decreased metabolites. Moreover, FMT from AOS-dosed mice improved specific blood metabolites involved in small intestinal functions such as aldosterone synthesis and secretion, fructose and mannose metabolism, galactose metabolism, fat digestion and absorption, cholesterol metabolism, carbohydrate digestion and absorption, and vitamin digestion and absorption, which indicated that FMT from AOS-dosed mice was superior in improving murine blood metabolism and small intestine function [38]. Furthermore, the correlation of gut microbiota and blood metabolites demonstrated that the “beneficial” microbe *Lactobacillales* assisted the recovery of blood metabolites [23], while the “harmful” microbe *Mycoplasmatales* did not. In summary, the data confirmed our hypothesis that FMT from a donor with superior microbiota had a more profound effect on small intestine function. FMT from AOS-dosed mice were more successful in improving small intestine function and blood metabolome than FMT from control mice. Our results suggest that gut microbiota from naturally produced AOS-treated donor could be used to prevent small intestinal mucositis induced by chemotherapeutics or other factors in recipients.

## Methods [detailed methods in supplementary information]

### Study design

All animal procedures were approved and conducted in accordance with the Qingdao Agriculture University Animal Care and Use Committee. Mice were maintained under a light:dark cycle of 12:12 h, at a temperature of 23 °C and humidity of 50%–70%; they had free access to food (chow diet) and water [23].

#### Part 1: AOS changed mouse small intestine microbiota

Three-week-old ICR male mice were injected a single dose of busulfan [40 mg/kg body weight (BW)] [23]. The following day, the mice were dosed with ddH_2_O as the control or AOS 10 mg/kg BW via oral gavage (0.1 ml/mouse/day). There were three treatment groups (30 mice/treatment): (1) vehicle control (ddH_2_O) designed as “A0” group; (2) busulfan alone (dosing with ddH_2_O) designated the “BA0” group; (3) busulfan plus AOS 10 mg/kg BW designated as the “BA10” group. AOS dosing solution was freshly made daily in ddH_2_O. Gavage dosing took place every morning for 2 weeks. After treatment, the mice were humanely terminated for the collection of intestinal microbiota samples for analysis.

#### Part 2: Mouse small intestine microbiota collection for the FMT experiment

Three-week-old ICR male mice were treated with ddH_2_O as the vehicle control or AOS 10 mg/kg body weight (BW), or AOS 100 mg/kg BW via oral gavage (0.1 ml/mouse/d). AOS dosing solution was freshly prepared on a daily basis and delivered every morning for 3 weeks. There were three groups (30 mice/treatment): (1) Control (ddH_2_O); (2) A10 (AOS 10 mg/kg BW); (3) A100 (AOS 100 mg/kg BW). After treatment, the mice were humanely euthanized to collect small intestine luminal contents (gut microbiota). The luminal contents from each group were pooled and homogenized, diluted 1:1 in 20% sterile glycerol (saline), and frozen. Before inoculation, small intestinal content samples were diluted in sterile saline to a working concentration of 0.05 g/ml and filtered through a 70-μm cell strainer (FMT).

#### Part 3: Gut microbiota transplants (FMT) [39]

Three-week-old ICR male mice were injected a single dose of busulfan [40 mg/kg body weight (BW)] [23]. The following day, the mice were dosed with saline as the control or FMT via oral gavage (0.1 ml/mouse/day). There were four treatment groups (30 mice/treatment): (1) Sa (injection 40 mg/kg BW of busulfan once [23] then dosed with saline); (2) Con-FMT [40 mg/kg BW busulfan once plus gut microbiota from control mice (Part 2)]; (3) A10-FMT [40 mg/kg BW busulfan once plus gut microbiota from AOS 10 mg/kg mice (Part 2)]; (4) A100-FMT [40 mg/kg BW busulfan once plus gut microbiota from AOS 100 mg/kg mice (Part 2)]. Recipient mice received oral FMT inoculations once daily for 1 week. The mice were then regularly maintained for another week (5 weeks of age) and then humanely euthanized to collect samples for different analyses.

### RNA isolation and RNA-seq analyses [37]

Briefly, total RNA was isolated using TRIzol Reagent (Invitrogen) and purified using a Pure-Link1 RNA Mini Kit (Cat: 12183018A; Life Technologies) following the manufacturers’ protocol. The library products were prepared for sequencing in an Illumina HiSeqTM 2500. The read number of each gene was transformed into RPKM (reads per kilo bases per million reads), and then differentially expressed genes were identified using the DEGseq package and the MARS (MA-plot-based method with random sampling model) method. Data were then analyzed by GO enrichment, KEGG enrichment, and multiple enrichment online (Metascape: http://metascape.org/gp/index.html#/ main/step1).

### Sequencing of microbiota from small intestine digesta samples and data analysis [37]

#### DNA Extraction

Total genomic DNA of the small intestine digesta was isolated using an E.Z.N.A.R Stool DNA Kit (Omega Bio-tek Inc., USA) following the manufacturer’s instructions.

#### Library preparation and sequencing

The V3-V4 region of the 16S rRNA gene was amplified using the primers MPRK341F (50-ACTCCTACGGGAGGCAGCAG-30) and MPRK806R (50-GGACTACHVGGGTWTCTAAT-30) with barcode. Then, the library was sequenced on the Illumina HiSeq 2500 platform and 300 bp paired-end reads were generated at the Novo gene.

#### Analysis of sequencing data

Operational taxonomic unit abundance information was normalized using a standard of sequence number corresponding to the sample with the least sequences. LEfSe was performed to determine differences in abundance.

### Plasma metabolite measurements using LC-MS/MS

Plasma samples were collected and immediately stored at −80 °C. Before LC-MS/MS analysis, the samples were thawed on ice and processed to remove proteins. Then samples were detected using ACQUITY UPLC and AB Sciex Triple TOF 5600 (LC/MS) as reported previously [23, 37].

### Histopathological analysis

Small intestine tissues were fixed in 10% neutral buffered formalin, paraffin-embedded, cut into 5 μm sections, and subsequently stained with hematoxylin and eosin (H&E) for histopathological analysis.

### Western blotting.

Western blotting analysis of proteins was carried out as previously reported [23, 37]. Briefly, small intestine tissue samples were lysed in RIPA buffer containing the protease inhibitor cocktail from Sangong Biotech, Ltd. (Shanghai, China). The primary antibodies (Abs) are listed in Supplementary Table 4.

### Detection of protein levels and location in the intestine using immunofluorescence staining

The methodology for immunofluorescence staining of small intestine samples is reported in our recent publications [23, 37]. Sections of intestine tissue (5 μm) were prepared and subjected to antigen retrieval and immunostaining as previously described.

### Statistical analysis

Data were analyzed using SPSS statistical software (IBM Co., NY, USA) with a one-way analysis of variance (ANOVA) followed by LSD multiple comparison tests. All groups were compared with each other for every parameter. The data were shown as the mean ± SEM. Statistical significance was based on *p* < 0.05. The correlation matrix between the gut microbiota and blood metabolites was generated using Pearson’s correlation coefficient [45].

## Supplementary information

**Additional file 1: Figure S1.** Small intestinal microbiota changes after 2-weeks of AOS dosing. The alpha index of the small intestine microbiota: a, Chao1 index; b, Shannon index.

**Additional file 2: Figure S2.** Additional data for RNA seq analysis. a, Immunofluorescence staining of Vil1 for small intestine samples. White arrows indicated the Vil1 staining in intestinal samples. b, Volcano plot for the expression of genes in Sa vs. Con-FMT. “no DEGs” means the non-differentially expressed genes. c, Volcano plot for the expression of genes in Sa vs. A10-FMT. d, Volcano plot for the expression of genes in Sa vs. A100-FMT. e, PCA analysis for gene expression of mouse intestine for Sa and Con-FMT groups. f, PCA analysis for gene expression of mouse intestine for Sa and A10-FMT groups. g, PCA analysis for gene expression of mouse intestine for Sa and A100-FMT groups. h, Venn plot shows the changed gene among Sa vs. Con-FMT, Sa A10-FMT, and Sa A100-FMT. i, KEGG enrichment analysis of the genes increased in Sa vs. Con-FMT in mouse small intestine samples. j, KEGG enrichment analysis of the genes increased in Sa vs. A10-FMT in mouse small intestine samples. k, KEGG enrichment analysis of the genes increased in Sa vs. A100-FMT in mouse small intestine samples.

**Additional file 3: Figure S3.** Small intestinal microbiota changes after FMT. The alpha index of the small intestine microbiota: a, Chao1 index; b, Shannon index.

**Additional file 4: Figure S4.** Additional data for blood metabolites. a, Correlation of the most changed metabolites in Sa vs. Con-FMT. b, Correlation of the most changed metabolites in Sa vs. A10-FMT. c, Correlation of the most changed metabolites in Sa vs. A100-FMT.

**Additional file 5: Data file 1.** Metabolite changes for mouse blood samples in the following comparisons: Sa vs. Con-FMT, Sa vs. A10-FMT, and Sa vs. A100-FMT.

**Additional file 6: Table S1.** Relative amounts of microbiota in small intestine samples after 2-weeks of AOS treatment.

**Additional file 7: Table S2.** Relative amounts of microbiota in small intestine samples after FMT.

**Additional file 8: Table S3.** The commonly changed blood metabolites in Sa vs. A10-FMT and A100-FMT.

**Additional file 9: Table S4.** Information for primary antibodies.

## Data Availability

RNA-seq raw data is deposited in NCBI’s Gene Expression Omnibus under accession number GSE137999. The microbiota raw sequencing data generated in this study has been uploaded to the NCBI SRA database with the accession number PRJNA 592378.
