# Supplementary figures and images for: Microbiota from alginate oligosaccharide-dosed mice successfully mitigated small intestinal mucositis
Source: Microbiome. 2020 Jul 25;8:112. doi: 10.1186/s40168-020-00886-x (PMC7382812; doi:10.1186/s40168-020-00886-x)

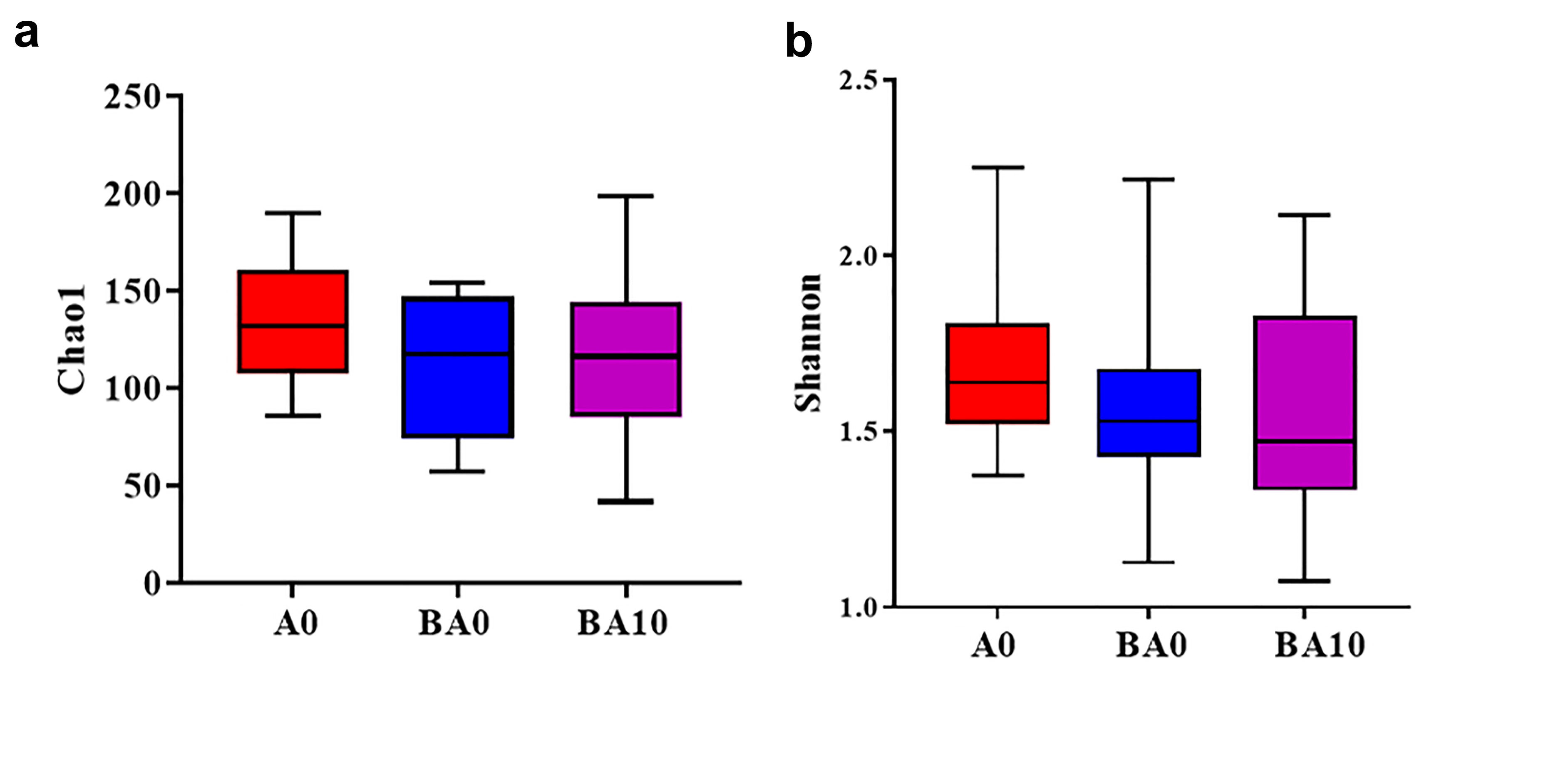

Supplement: Supplementary file 2 — Additional file 1: Figure S1. Small intestinal microbiota changes after 2-weeks of AOS dosing. The alpha index of the small intestine microbiota: a, Chao1 index; b, Shannon index. [file 40168_2020_886_MOESM1_ESM.tif]

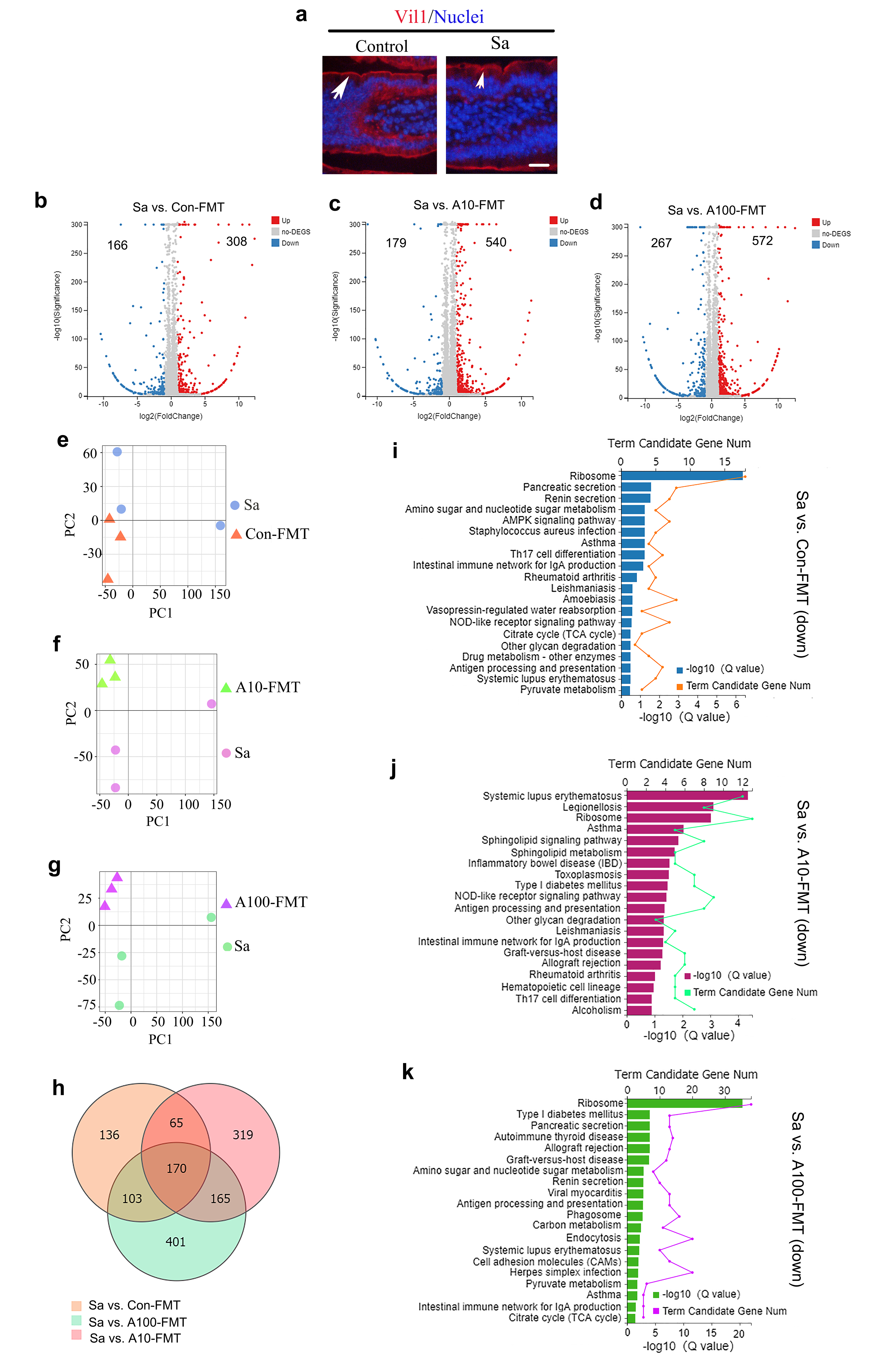

Supplement: Supplementary file 3 — Additional file 2: Figure S2. Additional data for RNA seq analysis. a, Immunofluorescence staining of Vil1 for small intestine samples. White arrows indicated the Vil1 staining in intestinal samples. b, Volcano plot for the expression of genes in Sa vs. Con-FMT. “no DEGs” means the non-differentially expressed genes. c, Volcano plot for the expression of genes in Sa vs. A10-FMT. d, Volcano plot for the expression of genes in Sa vs. A100-FMT. e, PCA analysis for gene expression of mouse intestine for Sa and Con-FMT groups. f, PCA analysis for gene expression of mouse intestine for Sa and A10-FMT groups. g, PCA analysis for gene expression of mouse intestine for Sa and A100-FMT groups. h, Venn plot shows the changed gene among Sa vs. Con-FMT, Sa A10-FMT, and Sa A100-FMT. i, KEGG enrichment analysis of the genes increased in Sa vs. Con-FMT in mouse small intestine samples. j, KEGG enrichment analysis of the genes increased in Sa vs. A10-FMT in mouse small intestine samples. k, KEGG enrichment analysis of the genes increased in Sa vs. A100-FMT in mouse small intestine samples. [file 40168_2020_886_MOESM2_ESM.tif]

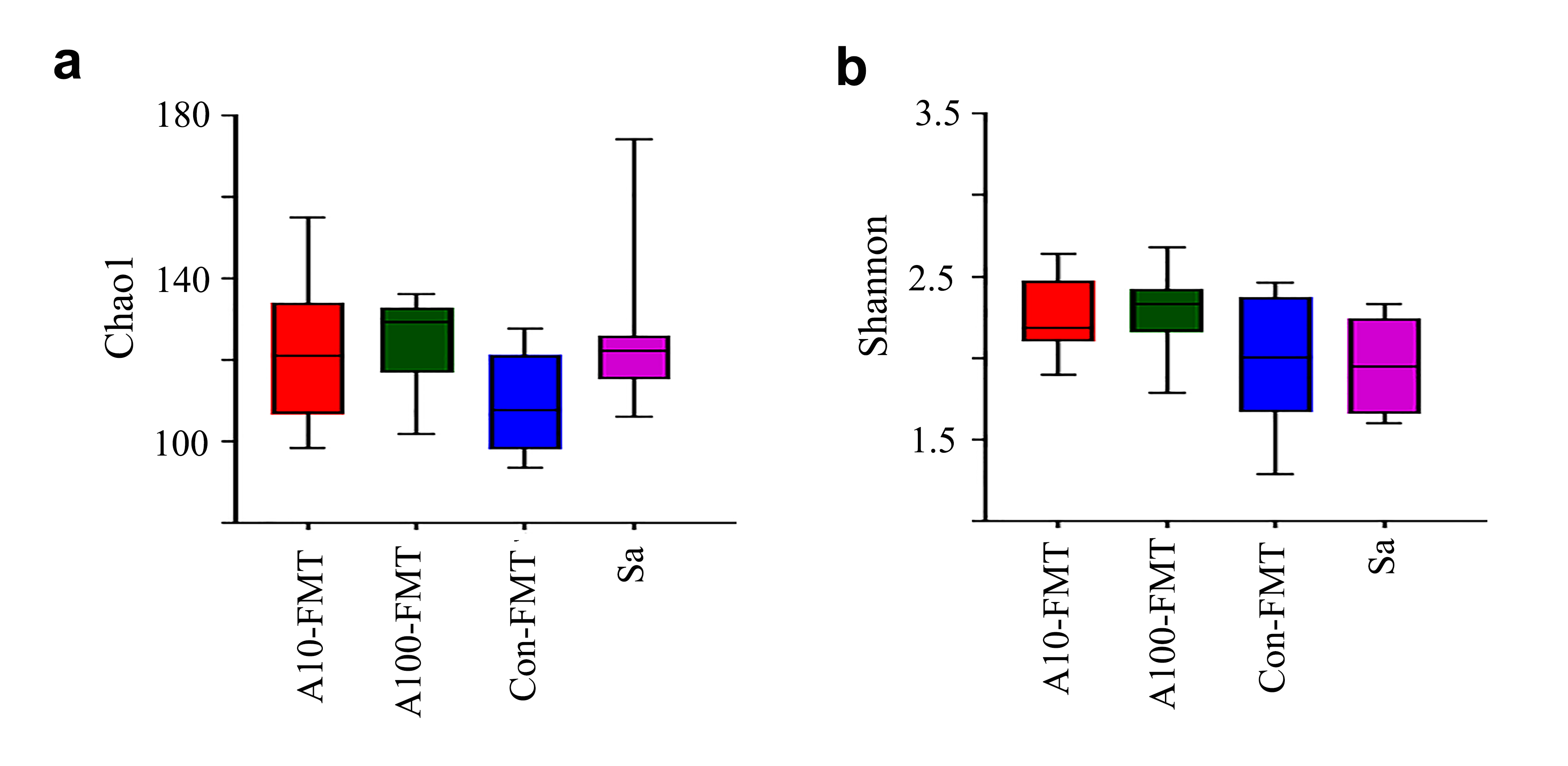

Supplement: Supplementary file 4 — Additional file 3: Figure S3. Small intestinal microbiota changes after FMT. The alpha index of the small intestine microbiota: a, Chao1 index; b, Shannon index. [file 40168_2020_886_MOESM3_ESM.tif]

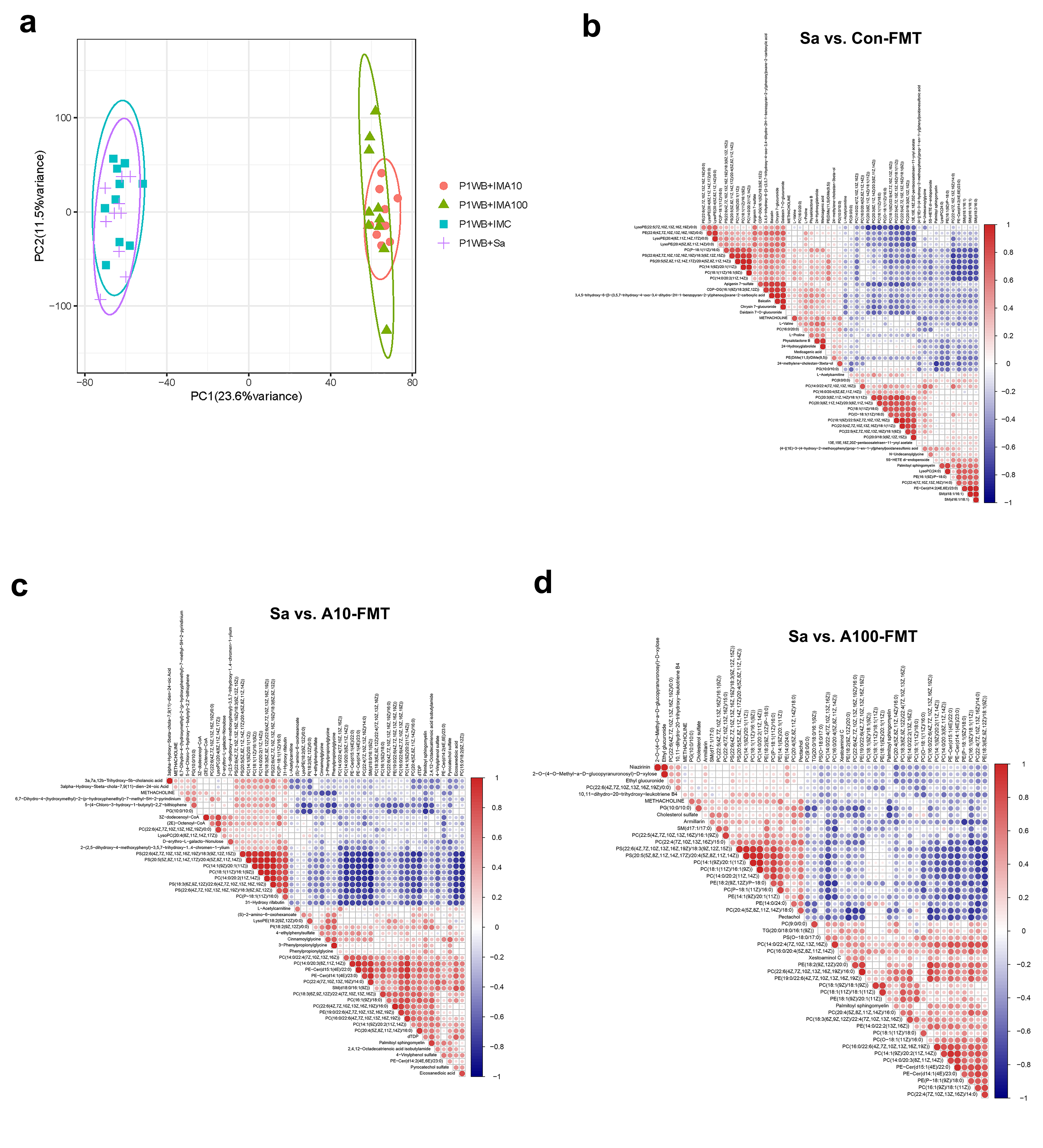

Supplement: Supplementary file 5 — Additional file 4: Figure S4. Additional data for blood metabolites. a, Correlation of the most changed metabolites in Sa vs. Con-FMT. b, Correlation of the most changed metabolites in Sa vs. A10-FMT. c, Correlation of the most changed metabolites in Sa vs. A100-FMT. [file 40168_2020_886_MOESM4_ESM.tif]
