# Supplementary material for: Microbiota from alginate oligosaccharide-dosed mice successfully mitigated small intestinal mucositis
Source: Microbiome. 2020 Jul 25;8:112. doi: 10.1186/s40168-020-00886-x (PMC7382812; doi:10.1186/s40168-020-00886-x)
Supplement: Supplementary file 10 — Additional file 9: Table S4. Information for primary antibodies. [file 40168_2020_886_MOESM9_ESM.docx]

Primary antibody information

| **Gene symbol** | **Name** | **Cat. #** | **Predicted size** | **Source (Animal)** | **Company** |
| --- | --- | --- | --- | --- | --- |
| Bcl-xl | Bcl-xl | bs-1336R | 26kd | Rabbit (polyclonal) | Beijing Biosynthesis Biotechnology CO. |
| Claudin | Claudin | bs-2183R | 22kd | Rabbit (polyclonal) | Beijing Biosynthesis Biotechnology CO. |
| Occludin | Rabbit Anti-Occludin antibody | bs-10011R | 59kDa | Rabbit (polyclonal) | Beijing Biosynthesis Biotechnology CO. |
| actin | actin | Ab3280 | 42kDa | Rabbit (polyclonal) | Abcam |
| p-PTEN | Phospho-PTEN | bs-3351R | 44kDa | Rabbit (polyclonal) | Beijing Biosynthesis Biotechnology CO. |
| Cx37 | Connexin37 | bs-4067R | 37kDa | Rabbit (polyclonal) | Beijing Biosynthesis Biotechnology CO. |
| GATA4 | GATA binding factor 4 | bs-23982R | 49kDa | Rabbit (polyclonal) | Beijing Biosynthesis Biotechnology CO. |
| Klf7 | Krueppel like factor 7 | bs-11865R | 33kDa | Rabbit (polyclonal) | Beijing Biosynthesis Biotechnology CO. |
| Catenin | Recombinant Anti-delta 1 Catenin/CAS antibody | Ab 92514 | 108kDa | Rabbit (monoclonal) | Abcam |
| SOX4 | SRY box containing gene 4 | bs-11208R | 52kDa | Rabbit (polyclonal) | Beijing Biosynthesis Biotechnology CO. |
| Vil1 | villin | ab130751 | 93kDa | Rabbit (monoclonal) | Abcam |
| E-cadherin | Anti-E Cadherin | ab11512 | 125kDa | Rat (monoclonal) | Abcam |

Zo-1 ZO1 tight junction protein Ab 190085 191kDa Goat (polyclonal) Abcam
